# Synergistic Effects of Curcumin and Antibiotics Against Drug-Sensitive and Multidrug-Resistant *Mycobacterium tuberculosis*

**DOI:** 10.3390/ijms262110414

**Published:** 2025-10-27

**Authors:** Jacqueline V. Lara-Espinosa, Jorge Barrios-Payán, Vasti Lozano-Ordaz, Dulce Mata-Espinosa, Enrique Becerril-Villanueva, María Dolores Ponce-Regalado, Rogelio Hernández-Pando

**Affiliations:** 1 Sección de Patología Experimental, Instituto Nacional de Ciencias Médicas y Nutrición Salvador Zubirán, Vasco de Quiroga 15, Belisario Domínguez Sección 16, Tlalpan, Ciudad de México 14080, Mexico or jacqueline.larae@incmnsz.mx (J.V.L.-E.); jorge.barriosp@incmnsz.mx (J.B.-P.); lozanoordazvasti@gmail.com (V.L.-O.); 2 Laboratorio de Psicoinmunología, Instituto Nacional de Psiquiatría Ramon de la Fuente Muñiz, Calzada México-Xochimilco 101, Colonia, Huipulco, Tlalpan, Ciudad de México 14370, Mexico; lusenbeve@imp.edu.mx; 3 Departamento de Ciencias de la Salud, Centro Universitario de los Altos, Universidad de Guadalajara, Av Rafael Casillas Aceves 120, Tepatitlán de Morelos 47620, Jalisco, Mexico; dorisponce61@hotmail.com

**Keywords:** tuberculosis, curcumin, adjunctive therapy, MDR-TB, antibiotic synergy

## Abstract

Tuberculosis (TB), caused by *Mycobacterium tuberculosis* (*Mtb*), remains a global health challenge, partly due to the prolonged duration and toxicity of standard antibiotic regimens. Adjunctive therapies that enhance antimicrobial efficacy and modulate host immunity are urgently needed. Curcumin, a natural bioactive compound derived from Curcuma longa, possesses broad therapeutic properties, including anti-inflammatory, antioxidant, antibacterial, and antiviral effects. This study evaluated the effects of curcumin in combination with first- and second-line antibiotics against *Mtb* in both in vitro and in vivo models. Our results demonstrated that curcumin exerts direct antibacterial activity against both the drug-sensitive H37Rv strain and a multidrug-resistant (MDR) clinical isolate. Furthermore, curcumin synergized with conventional antibiotics, enhancing bacterial clearance in infected macrophages while promoting the production of IL-12, a key cytokine in protective immune responses. In a murine model of progressive pulmonary TB, combination therapy with curcumin and first-line antibiotics significantly reduced the lung bacterial burden and improved behavioral outcomes compared to antibiotic treatment alone. These findings suggest that curcumin acts through both direct antimicrobial mechanisms and immune modulation, supporting its potential as an adjunctive therapy agent for TB. Future studies should focus on optimizing curcumin formulation, dosing, and bioavailability to facilitate the clinical translation of this compound.

## 1. Introduction

Due to the variety of chemical compounds found in plants, which may have significant therapeutic characteristics that can be utilized to treat a wide range of conditions, the value of plants has been acknowledged and documented since ancient times. Ancient cultures extensively utilized medicinal plants as a means of both prevention and treatment for a range of common and fatal diseases [1].

The perennial herbaceous plant *Curcuma longa*, also known as turmeric, is a member of the Zingiberaceae family [2]. It was first cultivated in India and is now widely grown in China, Sri Lanka, West and East Africa, and other tropical nations [3]. From the turmeric rhizomes, curcumin was isolated in 1815 by the researchers Vogel and Pelletier, and is one of the most essential isolated polyphenolic phytochemicals. Structurally, it is a linear diarylheptanoid composed of two aromatic rings connected by a seven-carbon chain with conjugated carbonyl and methoxy groups, which confer its characteristic yellow color and reactivity [4]. Curcumin offers a wide range of therapeutic applications, including the treatment of oxidative and inflammatory disorders, hyperlipidemia, rheumatoid arthritis, metabolic syndrome, and anxiety, and has hepatoprotective, and neuroprotective activities [4,5,6]. Recently, it has been demonstrated that curcumin exhibits antibacterial and antiviral activities [7].

Tuberculosis (TB) is an infectious disease caused by the bacterium *Mycobacterium tuberculosis* (*Mtb*), which primarily affects the lungs, leading to pulmonary TB. Additionally, all other organs and tissues, including lymph nodes, the brain, kidneys, and spine, can be affected [8]. TB is of great importance to humanity due to the magnitude of morbidity and mortality it has generated since the beginning of human civilization [9]. TB is preventable and typically treatable. However, after being supplanted by coronavirus disease (COVID-19) for three years, TB is likely to have regained its position as the world’s leading infectious agent-related cause of death in 2023 [9]. Every year, around 10 million people have TB, and since 2021, the number has been increasing [10]. Treatment of active TB requires 6 months of a combination of antibiotics (ABX), which is a significant obstacle to TB eradication [11].

MDR-TB, defined as infection with *Mtb* strains resistant to at least isoniazid (INH) and rifampicin (RIF), poses a major global health threat [12]. The rise of MDR-TB, and even extensively drug-resistant TB (XDR-TB), compromises the efficacy of existing treatment regimens, increases mortality, and threatens to reverse gains in TB control [13]. Therefore, the development of new anti-TB agents is urgent. Without new therapeutic options, the combination of rising resistance, limited access to care, extended and toxic regimens, and high mortality will continue [14,15]. New drugs with novel mechanisms, shorter and simpler treatment courses, improved tolerability, and broad access would help reduce transmission, enhance patient outcomes, and ultimately bring TB control and elimination within reach [16].

In TB research, curcumin is primarily explored as an adjunct, rather than a standalone therapy, to enhance immune response, reduce inflammation, decrease bacterial burden, or mitigate the side effects of TB drugs [17,18,19]. Previously, we observed that in BALB/c mice infected with *Mtb* (strain H37Rv), treatment with curcumin alone reduced lung bacillary load, decreased pneumonia, and also decreased neuroinflammation [19]. Although its potential as an adjunct therapy in infectious diseases has been increasingly recognized, its specific effects against *Mtb*, either alone or in combination with conventional ABX, remain insufficiently characterized. In particular, little is known about its ability to enhance macrophage-mediated bacterial clearance, its immunomodulatory activity in the context of TB infection, and its potential to reduce disease-associated lung pathology. Because of this, we aimed to evaluate the synergistic effect of curcumin in combination with conventional chemotherapy against TB.

This study aimed to investigate the effects of curcumin on *Mtb* both in vitro and in vivo. The in vitro analyses evaluated the direct anti-mycobacterial activity of curcumin against various *Mtb* strains, its potential synergistic interactions with standard ABX, its capacity to enhance the bactericidal activity of alveolar macrophages, and its immunomodulatory properties. For the in vivo experiments, a well-established murine model of chronic pulmonary TB was employed. Within this model, we assessed the impact of curcumin administered in combination with ABX on pulmonary bacillary burden and the extent of pneumonia-associated lung pathology. Furthermore, we examined the potential influence of the treatment on selected behavioral parameters of the animals.

## 2. Results

### 2.1. Antimicrobial Activity of Curcumin on Mtb

**Figure 1:**
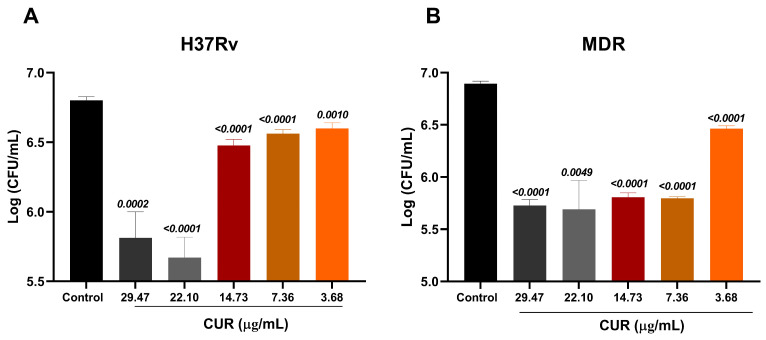
Antimicrobial activity of Curcumin on *Mtb.* Effect of Curcumin against the drug-sensitive *Mtb* H37Rv (**A**) and an MDR *Mtb* (**B**). For both strains, curcumin decreased the bacterial load from a concentration of 3.68 μg/mL. Unpaired *t*-test against the control.

We evaluated the antimicrobial activity of curcumin by determining the minimum inhibitory concentration (MIC). Our results demonstrate that curcumin possesses intrinsic antimicrobial activity against *Mtb*. We evaluated its effects at varying concentrations (3.68–29.47 μg/mL) on both the drug-sensitive H37Rv strain and an MDR clinical isolate by quantifying bacterial load in vitro. At 3.68 μg/mL, curcumin significantly reduced the bacterial burden of both H37Rv and MDR strains (colony-forming unit [CFU]) (Figure 1). Interestingly, the MDR strain exhibited a more pronounced reduction at 7.36 μg/mL, suggesting that higher curcumin concentrations may be required to overcome resistance mechanisms. Consequently, subsequent experiments with the MDR strain were conducted using 7.36 μg/mL. In contrast, no significant differences were observed among the 3.68–14.73 μg/mL concentrations in the H37Rv strain, indicating that even low doses are sufficient to achieve maximal antimicrobial effect in drug-sensitive bacteria; therefore, 3.68 μg/mL was chosen for all further assays with H37Rv. These findings highlight a differential dose–response pattern between drug-sensitive and resistant strains, underscoring the potential need for tailored curcumin dosing in adjunctive TB therapy.

### 2.2. Effect of Curcumin in Combination with First and Second-Line ABX Against Mtb

**Figure 2:**
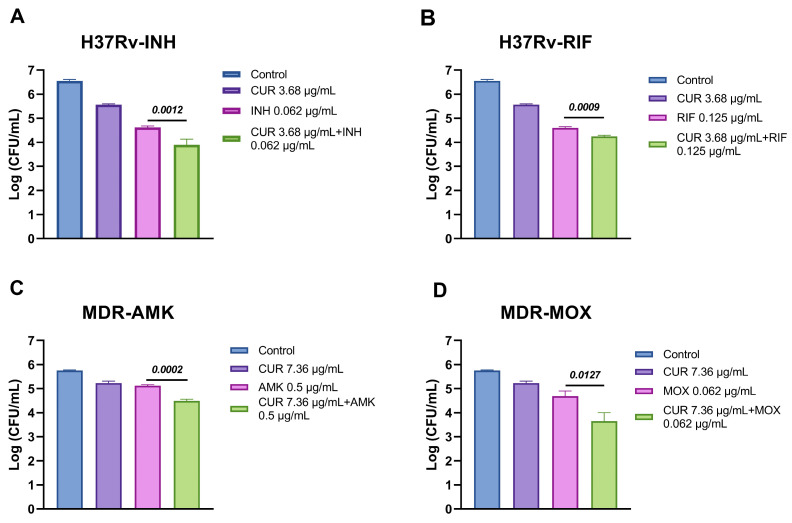
Synergistic effect of curcumin and ABX on *Mtb*. (**A**) Synergy between curcumin and INH against H37Rv. (**B**) Synergy between curcumin and RIF against H37Rv. (**C**) Synergy between curcumin and AMK against MDR. (**D**) Synergy between curcumin and MOX against MDR. Overall, the combination of curcumin with both first- and second-line ABX significantly reduced the bacillary load compared with ABX administered alone. Unpaired *t*-test against the antibiotic group.

After determining the MIC of curcumin against both H37Rv and MDR *Mtb* strains, we investigated its potential synergistic interactions with first- and second-line ABX. In the drug-sensitive H37Rv strain, curcumin exhibited an additive effect with INH and partial synergy with RIF, suggesting that curcumin can modestly enhance the efficacy of conventional therapy (Table 1). Notably, in the MDR strain, curcumin demonstrated partial synergy with amikacin (AMK) and moxifloxacin (MOX), indicating that it may help overcome resistance-associated limitations (Table 1). These interactions correlated with a noticeable reduction in CFU (Figure 2), providing functional evidence that curcumin could serve as a promising adjunctive agent in both drug-sensitive and drug-resistant TB treatment regimens.

### 2.3. Effect of Curcumin in Macrophages Infected with Mtb

**Figure 3:**
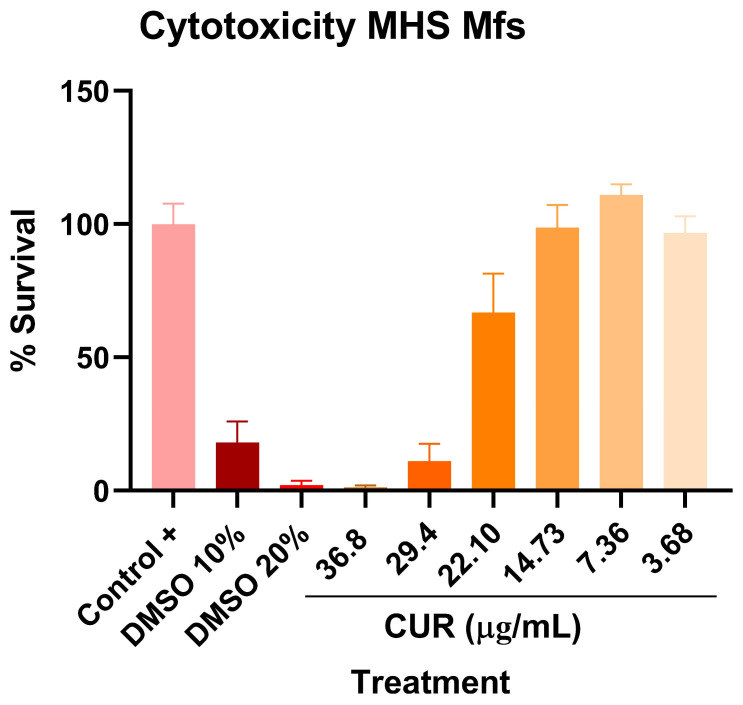
Determination of the cytotoxic effect of Curcumin on MH-S macrophages. The cytotoxic effect of different concentrations of curcumin (3.68–36.8 μg/mL) on uninfected MH-S macrophages was evaluated. Dimethyl sulfoxide (DMSO) at 10% and 20% in Roswell Park Memorial Institute medium (RPMI medium) was used as a positive cytotoxicity control. Curcumin was not toxic at concentrations ranging from 3.68 to 14.73 μg/mL.

**Figure 4:**
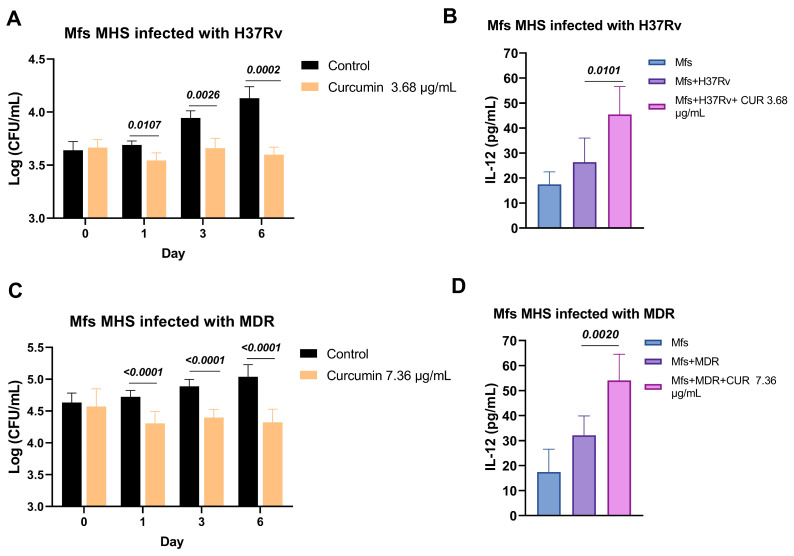
Effect of Curcumin on Macrophages Infected with *Mtb*. (**A**) Bacillary load of macrophages infected with H37Rv and treated with 3.68 μg/mL of curcumin. (**B**) IL-12 production by macrophages infected with H37Rv and treated with 3.68 μg/mL of curcumin. (**C**) Bacillary load of macrophages infected with MDR and treated with 7.36 μg/mL of curcumin. (**D**) IL-12 production by macrophages infected with MDR and treated with 7.36 μg/mL of curcumin. Curcumin reduced the intracellular load of bacteria in macrophages infected with H37Rv and MDR and increased the production of IL-12. Unpaired *t*-test against the control.

We next investigated the effects of curcumin on alveolar macrophages infected with *Mtb* H37Rv and MDR strains. Initially, we assessed the potential cytotoxicity of curcumin on uninfected macrophages, and no adverse effects were observed at concentrations ranging from 3.68 to 36.8 μg/mL (Figure 3). Subsequently, we examined its impact on the microbicidal activity of macrophages challenged with H37Rv and MDR strains. Treatment with curcumin enhanced the bactericidal capacity of macrophages against both strains, as evidenced by a significant reduction in intracellular bacillary load. This enhanced antimicrobial response was accompanied by an increase in Interleukin-12 (IL-12) production, suggesting that curcumin not only directly promotes bacterial clearance but also activates macrophage-protective anti-mycobacterial immune responses, potentially contributing to a more effective host defense against both drug-sensitive and drug-resistant *Mtb (*Figure 4).

### 2.4. Effect of Curcumin Administration in Conjunction with ABX on Advanced Pulmonary TB

**Figure 5:**
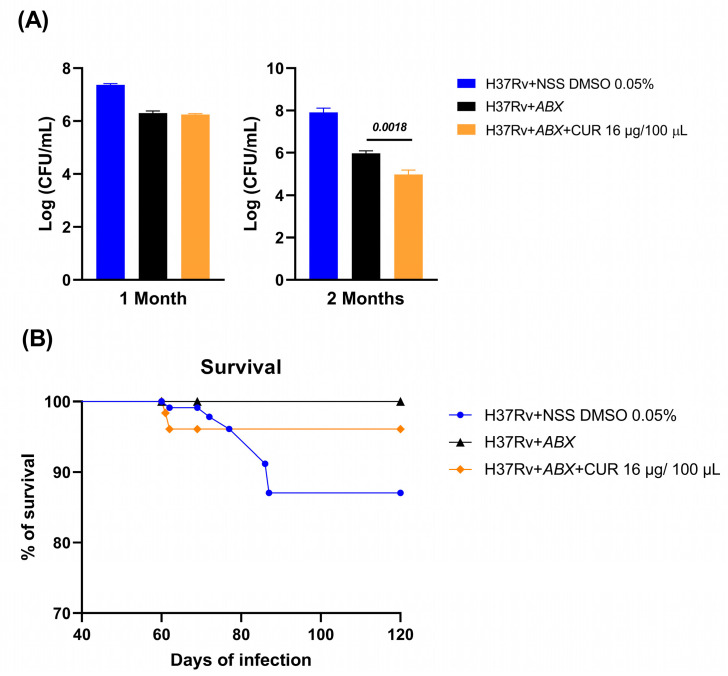
Effect of Curcumin Administration in Combination with ABX (RIF [10 mg/kg], INH [10 mg/kg], and PYZ [30 mg/kg]) on Advanced Pulmonary TB. (**A**) Bacillary burden in the lung after 1 and 2 months of treatment. (**B**) Animal survival. After two months of treatment, curcumin, in combination with ABX, reduced the bacterial load in the lungs. An infected control was used that received Normal Saline Solution (NSS) with 0.05% DMSO. Data are presented as mean ± Standard Error of the Mean (SEM). *t* test versus the control group receiving ABX. *n* = 12.

**Figure 6:**
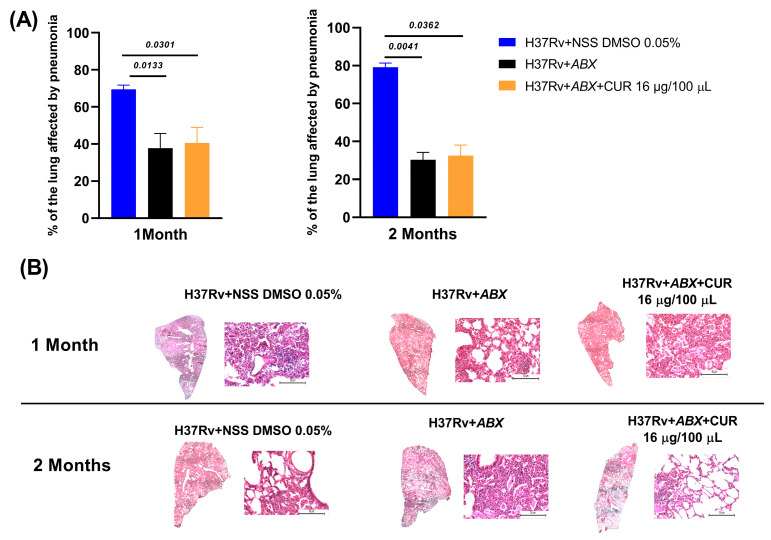
Effect of Curcumin Administration in Combination with ABX (RIF [10 mg/kg], INH [10 mg/kg], and PYZ [30 mg/kg]) on Pneumonia in Advanced Pulmonary TB. (**A**) Histological analysis of lungs from mice infected with H37Rv and treated with curcumin and ABX for one and two months, expressed as the percentage of lung surface area affected by pneumonia. (**B**) Representative micrographs of whole lungs and a representative 20X area after one and two months of curcumin administration. An infected control was used that received NSS with 0.05% DMSO. Data are presented as mean ± SEM. *t*-test against the control that received ABX. *n* = 12.

We evaluated the effects of curcumin in combination with *ABX* (RIF [10 mg/kg], INH [10 mg/kg], and pyrazinamide (PYZ) [30 mg/kg]) in mice infected with *Mtb* H37Rv. Treatment was initiated on day 60 post-infection, and bacillary load was measured at 1 and 2 months. At the 1-month time point, the addition of curcumin did not enhance the bactericidal efficacy of *ABX*, suggesting that short-term co-administration may be insufficient to impact bacterial clearance. However, after 2 months, the curcumin–antibiotic combination produced a more pronounced reduction in bacillary burden compared with *ABX* alone, indicating a time-dependent synergistic effect (Figure 5). Despite this enhanced bacterial clearance, no significant differences in survival were observed between groups, suggesting that factors beyond bacillary load, such as systemic pathology or host immune status, may govern mortality under these experimental conditions (Figure 4). Similarly, the extent of pneumonia-associated lung pathology remained comparable across groups at both time points (Figure 6), implying that while curcumin may potentiate bacterial clearance, it does not substantially mitigate TB-induced pulmonary inflammation within the studied timeframe.

### 2.5. Effect of Curcumin Administration in Combination with ABX on the Sickness Behavior of Mice with Advanced TB

**Figure 7:**
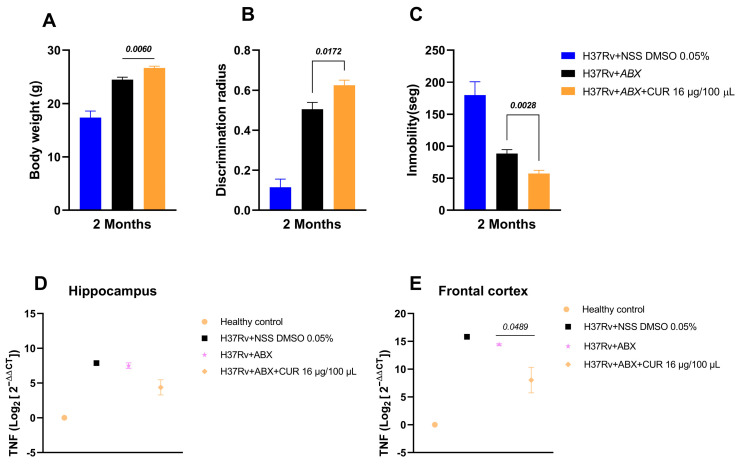
Effect of curcumin in combination with ABX (RIF [10 mg/kg], INH [10 mg/kg], and PYZ [30 mg/kg]) on behavioral parameters and inflammation. (**A**) Body weight. (**B**) Short-term memory. (**C**) Depression like behavior. (**D**) TNF expression in the hippocampus. (**E**) TNF expression in the frontal cortex. The combination of curcumin and ABX improved body weight and short-term memory in the animals. It decreased depressive-like behavior and TNF expression in the hippocampus and frontal cortex. An infected control was used that received NSS with 0.05% DMSO. Data are presented as mean ± SEM. *t*-test against the control that received ABX. *n* = 12.

Administration of curcumin combined with ABX influenced both behavioral and neuroinflammatory outcomes in mice with advanced pulmonary TB. After 2 months of treatment, animals receiving the curcumin–antibiotic combo showed slightly higher body weight than antibiotic-only controls, suggesting a reduction in infection-related sickness behavior. Significantly, the combination treatment also decreased depression-like behavior in the tail suspension test and improved short-term memory at 2 months, suggesting that curcumin might offer neuroprotective effects during chronic TB infection (Figure 7).

At the molecular level, analysis of Tumor Necrosis Factor (TNF) expression in the hypothalamus, hippocampus, and frontal cortex revealed that curcumin–antibiotic treatment modestly decreased TNF levels in the hippocampus and frontal cortex compared with controls, consistent with reduced neuroinflammation. Together, these findings indicate that curcumin not only potentiates the behavioral recovery of TB-infected mice but may also mitigate infection-associated neuroinflammatory responses, supporting its potential as an adjuvant therapy to improve both systemic and central nervous system outcomes in chronic TB (Figure 7).

## 3. Discussion

Despite advances in treatment, TB continues to cause significant morbidity and mortality worldwide. The emergence of MDR and XDR *Mtb* strains has severely compromised the effectiveness of standard antibiotic regimens, emphasizing the urgent need for new therapeutic strategies. Standard anti-TB treatment remains lengthy and often produces adverse effects that can lead to poor adherence and suboptimal outcomes [10]. In this context, host-directed therapies or adjunctive agents, including immunomodulators and phytochemicals, may enhance bacterial clearance, modulate inflammation, and reduce tissue damage when combined with first- or second-line ABX [20,21].

Our findings with curcumin support this approach. The compound not only decreased bacterial burden in infected macrophages and murine lungs but also exhibited immunomodulatory effects that complemented conventional therapy. These results strengthen the rationale for integrating adjunctive compounds into TB treatment regimens to improve efficacy, reduce toxicity, and potentially shorten therapy duration.

A key observation from our work is that curcumin’s activity aligns with previous evidence of synergistic effects with ABX. Marini et al. (2018) demonstrated that curcumin synergized with amikacin, ciprofloxacin, clarithromycin, and linezolid against *M. abscessus*, reducing bacterial growth and biofilm formation [22]. Similarly, Tousif et al. (2017) reported that nanocurcumin combined with INH enhanced bacterial clearance, prevented reactivation, promoted a Th1-type immune response, and lessened INH-induced hepatotoxicity [23]. These studies, together with our results, suggest that curcumin can potentiate the efficacy of both first- and second-line anti-TB drugs while mitigating their toxic effects.

At the cellular level, curcumin enhances macrophage-mediated clearance of *Mtb*. Bai et al. (2016) showed that pretreatment of THP-1 monocytes and human alveolar macrophages with curcumin reduced intracellular bacterial load by inducing apoptosis and autophagy [17]. Comparable results were obtained in RAW 264.7 macrophages infected with MDR clinical isolates, confirming that curcumin facilitates bacterial clearance [24]. These findings support a dual mechanism of action—direct antimicrobial activity combined with activation of host defense pathways.

The immunomodulatory properties of curcumin further expand its therapeutic potential. Ahmad et al. (2019) demonstrated that curcumin nanoparticles promote autophagy in antigen-presenting cells, increase pro-inflammatory cytokine secretion, and enhance BCG vaccine efficacy by expanding Th1- and Th17-type central memory T cells [25]. In line with these observations, we found that curcumin increased IL-12 production by macrophages. IL-12 plays a central role in macrophage responses against *Mtb* by stimulating IFN-γ secretion from NK and T cells, which enhances macrophage bactericidal activity and nitric oxide production [26,27]. This capacity to regulate immune activation while limiting inflammation suggests that curcumin can protect tissues from excessive immune-mediated damage.

Interestingly, our results also demonstrate that curcumin exerts anti-inflammatory effects in the brain. Neuroinflammation may result from peripheral immune activation and oxidative stress, leading to microglial activation and neuronal injury [28]. We observed that curcumin in combination with ABX significantly reduced TNF mRNA expression in the hippocampus and frontal cortex compared with ABX-TB controls, indicating a potent anti-inflammatory effect within the central nervous system. These findings are consistent with previous studies showing that curcumin attenuates neuroinflammation and oxidative damage across several neuropathological conditions [29]. Experimental data further indicate that curcumin modulates oxidative–nitrosative stress and downregulates TNF, IL-1, Nuclear Factor kappa B (NFκB), and caspase-3 in various brain regions [30]. In ischemic and neurodegenerative models, curcumin decreases IL-23, IL-17, Cyclooxygenase-2 (COX-2), and Prostaglandin E_2_ (PGE_2_) through the Peroxisome Proliferator-Activated Receptor Gamma (PPARγ) activation and inhibition of TLR-2/4–NFκB signaling, thereby preventing neuronal damage [31,32].

Beyond its anti-inflammatory role, curcumin improved behavioral outcomes in TB-infected mice. Cytokine-induced neuroinflammation has been implicated in the pathophysiology of depression and other neuropsychiatric disorders due to its effects on oxidative stress, neurotransmitter regulation, and neuronal survival [33]. Consistent with this, the combination of curcumin and ABX reduced sickness behavior and depression-like behaviors, and improved short-term memory in TB-infected mice. Similar behavioral and neurochemical improvements have been described in animal models of stress and metal toxicity, where curcumin increased antioxidant enzyme activity, locomotor performance, learning, and monoamine levels [34,35]. In humans, curcumin supplementation has also shown anxiolytic and antidepressant effects associated with its antioxidant, anti-inflammatory, and dopaminergic properties, as well as modulation of neurotrophic factors such as brain-derived neurotrophic factor (BDNF) [36,37].

Taking together, these findings suggest that curcumin provides a multidimensional therapeutic benefit in TB, not only enhancing antibiotic efficacy and immune responses but also attenuating neuroinflammation and improving behavioral outcomes.

Despite this growing body of evidence, several important limitations remain. Curcumin suffers from poor solubility and very low bioavailability, which limit its systemic distribution and therapeutic potential. These physicochemical constraints result in poor absorption, rapid metabolism, and low plasma concentrations following administration. Advances in formulation strategies—such as encapsulation in polymeric or lipid nanoparticles, liposomes, and co-administration with bioenhancers like piperine—have improved its pharmacokinetic profile [25], but optimal dosing, administration routes, and tissue distribution remain undefined. Moreover, most available data, including our own findings, derive from in vitro and murine models. While valuable for understanding host–pathogen interactions, these models have inherent translational limitations due to differences in immune and metabolic responses between rodents and humans. Future studies should include detailed pharmacokinetic and pharmacodynamic analyses, as well as alternative models such as guinea pigs or non-human primates that more accurately mimic human TB.

In addition, potential drug–drug interactions between curcumin and standard anti-TB agents should be investigated, as curcumin is known to modulate cytochrome P450 enzymes and efflux transporters, which could affect antibiotic metabolism and efficacy [7]. Well-designed clinical trials are ultimately required to confirm curcumin’s safety, bioavailability, and synergistic or immunomodulatory effects in humans, and to determine whether the promising preclinical results can be translated into tangible clinical benefits.

In summary, our findings, together with previous evidence, highlight curcumin as a promising adjunctive agent for TB therapy. Curcumin exhibits direct antimycobacterial activity, synergizes with first- and second-line ABX, enhances macrophage-mediated clearance, and exerts anti-inflammatory and neuroprotective effects that contribute to improved immune balance and behavioral recovery. Addressing its pharmacokinetic limitations and validating its efficacy in clinical trials will be crucial next steps. In the near term, curcumin appears best suited as a complementary therapy to existing anti-TB regimens, with the potential to enhance efficacy, reduce drug toxicity, and improve both systemic and neurological outcomes associated with chronic infection.

## 4. Materials and Methods

### 4.1. Experimental Strategy

**Figure 8:**
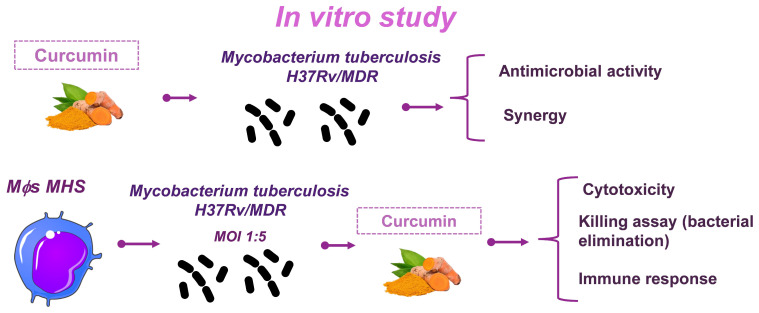
Experimental strategy of the in vitro study. In the in vitro phase, the effect of curcumin on the growth of *Mtb* H37Rv and MDR was evaluated by determining the MIC. We then assessed its synergistic effect with first- and second-line ABX. We then evaluated its effect on infected macrophages, determining bacillary load and immunomodulation. Parts of the figure were drawn using pictures from Servier Medical Art. Servier Medical Art by Servier is licensed under a Creative Commons Attribution 3.0 Unported License (https://creativecommons.org/licenses/by/3.0/, accessed on 8 August 2025).

**Figure 9:**
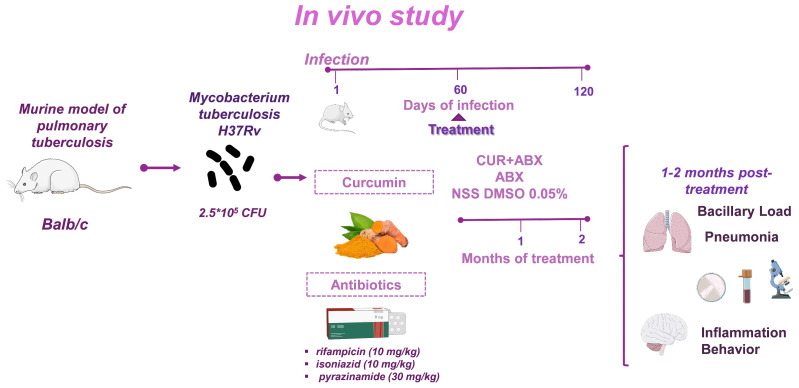
Experimental strategy of the in vivo study. In the in vivo phase, we evaluated the effect of combining curcumin with first-line ABX (RIF [10 mg/kg], INH [10 mg/kg], and PYZ [30 mg/kg]) in a progressive TB model. Bacterial burden, lung damage, neuroinflammation, and behavioral changes were determined after 1 and 2 months of treatment. Parts of the figure were drawn using pictures from Servier Medical Art. Servier Medical Art by Servier is licensed under a Creative Commons Attribution 3.0 Unported License (https://creativecommons.org/licenses/by/3.0/, accessed on 8 August 2025).

This study was conducted in two phases. In the in vitro phase, we evaluated the effect of curcumin on the growth of *Mtb* H37Rv and MDR strains by determining the MIC. Its potential synergistic activity with first- and second-line ABX was also assessed. Subsequently, we examined the effect of curcumin on infected macrophages by measuring bacillary load and immunomodulatory responses (Figure 8). In the second phase of the study, we investigated the effect of combining curcumin with first-line ABX (RIF [10 mg/kg], INH [10 mg/kg], and PYZ [30 mg/kg]) in a progressive TB model. After 1 and 2 months of treatment, we evaluated the bacterial burden, lung pathology, neuroinflammation, and behavioral alterations. Animals were monitored daily, and those exhibiting signs of respiratory insufficiency, severe cachexia, or complete immobility were humanely euthanized under pentobarbital anesthesia (Figure 9).

### 4.2. Preparation of Curcumin

Curcumin (CAS 458-37-7, Sigma-Aldrich, St. Louis, MO, USA) was dissolved in sterile water or RPMI medium containing 0.05% DMSO. For in vitro assays, concentrations ranging from 3.68–36.8 μg/mL were used. For the in vivo experiments, after 60 days of infection, groups of six mice (per sacrifice, in two independent experiments) received intraperitoneal injections of curcumin (16 µg in 100 µL) three times per week. Control groups were administered 100 µL NSS (9% NaCl) with 0.05% DMSO.

### 4.3. Determination of the Antimicrobial Activity of Curcumin on Mtb In Vitro

To evaluate the antimicrobial effect of Curcumin on *Mtb*, the drug-sensitive strain H37Rv and the MDR isolate (CIBIN/UMF). The clinical isolate, derived from a patient with severe pulmonary TB, was characterized at the Centro de Investigación Biomédica del Noreste, Instituto Mexicano del Seguro Social. The isolate was resistant to streptomycin, INH, RIF, ethambutol, and PYZ [38]. For both strains, we initiated cultures with 1 mL of bacterial solution in 6 mL of 7H9 medium (Middlebrook, Sigma-Aldrich, St. Louis, MO, USA) supplemented with Oleic, Albumin, Dextrose, and Catalase (OADC [Middlebrook, Becton, Dickinson and Company, Sparks, MD, USA]) and incubated at 37 °C with shaking for 14 days. After 14 days, the optical density of each bacterium was adjusted to 0.05 at 600 nm. 100 μL of the bacterial suspension was placed in 96-well plates, followed by 100 μL of different curcumin concentrations (3.68–29.47 μg/mL). We had bacterial growth control, in which we added 100 μL of 7H9 medium. The plates were placed at 35 °C with shaking at 70 rpm for 7 days for H37Rv and 10 days for MDR. After the incubation time, we determined the CFU of each concentration studied using 1:10 serial dilutions. Each dilution was seeded on 7H10 Agar Base plates (Middlebrook, Becton, Dickinson and Company, Sparks, MD, USA) and incubated at 37 °C for 21 days, after which they were counted.

### 4.4. Determination of the Combined Antimicrobial Activity of Curcumin with First and Second-Line ABX

To determine the synergy activity of curcumin with first- and second-line ABX in *Mtb*, a checkerboard assay was performed. For this, a culture of *Mtb* H37Rv or MDR, prepared as mentioned above, was briefly adjusted to 0.05 OD at 600 nm. In a 96-well plate, 50 μL of 7H9 medium was placed per well. 50 μL of a solution containing four times the maximum concentration of curcumin to be tested was placed in triplicate with 50 μL of medium. The contents of the wells were mixed appropriately. Then 50 μL was transferred to the next well, performing five serial dilutions (dilution factor 1:2). Subsequently, 50 μL of a solution containing four times the half MIC (0.5 MIC) previously determined for RIF (0.0625 μg/mL), INH (0.03125 µg/mL), AMK (0.25 µg/mL), and MOX (0.03125 µg/mL) was added to each dilution. Finally, the previously prepared bacterial suspension was shaken and sonicated for 45 s to place 100 μL per well. Controls for bacterial growth and no-growth conditions were established by placing 100 μL of the bacterial suspension into 100 μL of 7H9 medium and 200 μL of 7H9 medium, respectively. An antibiotic-only control was also added at a concentration of 0.5 MIC. The plate was left shaking at 35 °C for 7 and 10 days for H37Rv and MDR, respectively; subsequently, the CFU were counted as mentioned previously. The data produced by the checkerboard assay were analyzed in terms of the FICI, comparing the MIC value of each agent alone with the MIC value derived from the combination. The FICI is a parameter used to evaluate the interaction between two antimicrobial agents. It is calculated from the MIC values of each agent alone and in combination [39]. It was calculated as indicated in the formula [39]:

(1) $$\mathrm{FICI}=\frac{MIC\;A\;in\;combination}{MIC\;A\;in\;alone}+\;\frac{MIC\;B\;in\;combination}{MIC\;B\;in\;alone}$$

### 4.5. Evaluation of the Effect of Curcumin on Macrophage Infection and Bacillary Load Assay

To evaluate the effect of curcumin on macrophages infected with *Mtb*, the murine alveolar macrophage cell line MH-S (CRL-2019, ATCC, Manassas, VA, USA) was used. Cells were cultured in RPMI medium (RPP12, Caisson Labs, Smithfield, UT, USA) supplemented with 10% fetal bovine serum (FBS) (26140-079, Thermo Fisher Scientific, Waltham, MA, USA) and maintained at 37 °C in a humidified incubator with 5% CO_2_. Initially, the cytotoxicity of different curcumin concentrations was assessed in uninfected MH-S cells using the resazurin (AlamarBlue, Hercules, CA, USA) assay. DMSO at 10% and 20% in RPMI medium was used as a positive cytotoxicity control. For infection experiments, MH-S cells were seeded one day prior to infection in 96-well culture plates (SC-204444, Santa Cruz Biotechnology Inc., Dallas, TX, USA) at a density of 10,000 cells per well and incubated overnight under standard culture conditions. Adherent cells were then infected with *Mtb* H37Rv or MDR strains at a multiplicity of infection (MOI) of 1:5, suspended in RPMI medium, and incubated for 3 h at 37 °C. Following infection, cells were washed three times with RPMI medium containing 1% streptomycin (MP 1670049) to remove extracellular bacilli. Fresh RPMI medium supplemented with 10% FBS was added, and cells were further incubated at 37 °C for 1, 3, or 6 days. At each time point, infected macrophages were lysed in 7H9 medium containing 0.1% sodium dodecyl sulfate (SDS), incubated for 10 min, and then 20% bovine serum albumin was added. Serial dilutions of the lysates were prepared in 7H9 medium and plated onto Middlebrook 7H10 agar plates. The CFU were enumerated after 21 days of incubation.

Supernatants from macrophages infected with H37Rv or MDR strains and treated for 6 days with 3.68 μg/mL or 7.36 μg/mL of curcumin were collected, centrifuged at 14,000 rpm for 1 min, and immediately stored at −70 °C until analysis. Secreted cytokine levels were quantified following the manufacturer’s instructions using the Mouse Cytokine Magnetic 20-Plex Panel kit (LMC0006M, Life Technologies/Invitrogen, Carlsbad, CA, USA). Measurements were performed on a Bio-Plex 200 system (Bio-Rad, Hercules, CA, USA) at the Red de Apoyo a la Investigación (RAi) of the National Institute of Medical Sciences and Nutrition Salvador Zubirán (INCMNSZ, Mexico City, Mexico). Cytokine concentrations were automatically calculated using the dedicated software provided with the Bio-Plex 200 system.

### 4.6. Use of Animals

A total of 80 male BALB/c mice were used for the in vivo experiments. The sample size for this study was determined based on the principles of the 3Rs (Replacement, Reduction, and Refinement) to ensure the ethical and responsible use of laboratory animals. The animals were distributed in groups of 24 mice per treatment as indicated in Table 2. Eight more mice were infected due to deaths that occurred in the groups. Efforts were made to minimize the number of animals used while maintaining sufficient statistical power to detect meaningful differences. All experimental procedures were conducted in accordance with the Animal Research: Reporting of In Vivo Experiments (ARRIVE) guidelines and approved by the Institutional Animal Care and Use Committee of the INCMNSZ. The study design and handling procedures were intended to minimize animal pain, distress, and discomfort. To achieve this, animals were anesthetized using sevoflurane inhalation during invasive procedures, and pentobarbital was administered for euthanasia. Handling was performed by trained personnel to reduce stress, and animals were housed under controlled environmental conditions (temperature, humidity, and light/dark cycles) with free access to food and water. Continuous monitoring was performed to ensure animal welfare throughout the study.

### 4.7. The Murine Model of Pulmonary TB

The murine model of pulmonary TB has been previously described [40]. Mice were infected with the reference strain H37Rv, which was cultured in 7H9 selective medium supplemented with OADC [40]. Prior to infection, animals were anesthetized with 2% sevoflurane vapor and then intratracheally inoculated with 2.5 × 10^5^ CFU of *Mtb* in 100 µL of NSS. Following inoculation, mice were maintained in an upright position until normal reflexes and muscle tone were restored. Animals were housed in groups of six per cage within microisolators connected to negative-pressure regulators, all located in a biosafety level 3 (BSL-3) animal facility. Survival was monitored continuously throughout the experimental period. The researcher in charge of infecting the animals randomly distributed them into cages containing six mice. Treatment was randomly assigned to groups of 6 mice per cage. To avoid problems of confusion in treatments, each cage contains information on the indicated treatment.

### 4.8. Treatment with Curcumin and First-Line ABX

To minimize animal use, the in vivo part was conducted only with the H37Rv strain. Mice infected with H37Rv received curcumin (16 μg/100 μL) treatments in conjunction with the first-line therapy for the drug-sensitive H37Rv strain. The ABX included RIF (10 mg/kg), INH (10 mg/kg), and PYZ (30 mg/kg), all dissolved in sterile NSS. Each mouse was given 100 μL of the antibiotic mixture orally from Monday to Friday. This treatment protocol mirrors that used in humans, and the doses were based on three separate experiments previously conducted with our model [41,42].

### 4.9. CFU Methodology for Determining Pulmonary Bacillary Loads

In two independent trials, the right lung from six mice was collected for bacterial colony enumeration. Tissues were homogenized in sterile tubes containing 1 mL of Phosphate-Buffered Saline (PBS)-tween 80 0.05% using a FastPrep homogenizer (MP Biomedicals, Navi Mumbai, Maharashtra). The homogenates were suspended in 7H9 medium, serially diluted, and plated onto Middlebrook 7H10 agar. CFU were counted after 21 days of incubation, and results were expressed as log_10_ CFU/mL.

### 4.10. Preparation of Lung Tissue for Morphometric Analysis

The left lungs of six mice from each treatment group in two independent experiments were fixed with 2 mL of absolute ethanol, perfused intratracheally, and then immersed in 30 mL of absolute ethanol for 72 h. The lungs were dehydrated using the histological technique and then embedded in paraffin blocks. Sagittal sections of 5 μm thickness were made. The sections were stained with hematoxylin and eosin (H&E) for morphometric analysis. The morphometric study was performed using a digital light microscope, an image analyzer (Q-Win Leica 500, Leica Microsystems, Milton Keynes, UK) and a camara (Olympus DP70 camera, Olympus Corporation, Tokyo, Japan) from a set of micrographs (taken with the 20× objective) of the entire lung section, corresponding to 100% of the lung area. We determined the percentage of the lung surface affected by pneumonia. Measurements were made blindly with respect to the experimental treatment to which each lung belonged.

### 4.11. Behavioral Studies

The behavioral testing methodology for the murine model of progressive TB has been described previously [43]. Animals were habituated to the testing environment 24 h before each assessment. To avoid repeated exposure effects, each mouse was evaluated only once at the designated post-treatment time points. All behavioral tests were conducted during the first four hours of the dark phase of the light cycle, and a blinded evaluator recorded observations.

Following 1 and 2 months of treatment, sickness behavior was assessed by measuring weight loss. Body weight was measured at each time point, and weight loss in *Mtb*-infected mice was expressed as a percentage of body weight change. Depression-like behavior was assessed using the tail suspension test [44]. Mice were suspended by the tail for 6 min, and immobility time (reflecting behavioral despair) was recorded. Short-term memory was evaluated using the Object Recognition Test [45]. During habituation, mice explored an open field for 10 min. After 24 h, two identical objects (A) were placed in the arena for 3 min. Short-term memory was assessed 30 min later by replacing one familiar object (A) with a novel object (B), and interactions with each object (sniffing or touching with the forepaws) were recorded over 3 min. Results were expressed as the discrimination ratio, calculated as the percentage of interactions with the novel object relative to total object interactions.

### 4.12. Reverse Transcription Polymerase Chain Reaction (RT-PCR) Analysis of TNF Expression

mRNA from the hippocampus and frontal cortex of six control and infected mice per time point was extracted using the RNeasy Mini Kit (Qiagen GmbH, Hilden, Germany). RNA quality and quantity were verified by spectrophotometry (260/280) and gel electrophoresis. cDNA was synthesized from 100 µg RNA with oligo(dT) primers using the Omniscript kit (Qiagen GmbH, Hilden, Germany). Real-time PCR was performed using QuantiTect SYBR Green Master Mix (Qiagen, Venlo, The Netherlands) on a 7500 RT-PCR system (Applied Biosystems, Waltham, MA, USA) with primers for *Glyceraldehyde-3-phosphate dehydrogenase* (*GAPDH*) and *TNF* (Table 3). Cycling conditions were 95 °C for 15 min, followed by 40 cycles of 95 °C for 20 s, 60 °C for 20 s, and 72 °C for 34 s. Samples were run in duplicate, and relative expression was calculated using the 2^−ΔΔCt^ method [46]. The results were presented as log_2_ of the 2^−ΔΔCt^.

### 4.13. Statistics Analysis

In vitro results are expressed as mean ± Standard Deviation (SD) from four independent experiments conducted in triplicate. In vivo data are shown as mean ± SD or SEM from six mice across two independent experiments. Normality was tested using the Shapiro–Wilk test. Unpaired *t*-tests were used to determine statistical significance between the experimental groups and their respective controls. A *p*-value of less than 0.05 was considered significant. Analyses were conducted with GraphPad Prism v9.4.1 (GraphPad, San Diego, CA, USA).

## 5. Conclusions

Curcumin enhances the antibacterial efficacy of first- and second-line ABX against both drug-sensitive and MDR *Mtb* through direct antimicrobial activity and immunoregulation, including increased IL-12 production. In vitro and in vivo results demonstrate that combination therapy reduces bacterial burden and improves host outcomes, highlighting curcumin’s potential as a safe and effective adjunctive agent in TB treatment.

## Figures and Tables

**Table 1:** Fractional Inhibitory Concentration Index (FICI) from the combination of curcumin with first-line ABX and second-line ABX.

Mtb	Compound	MIC	CUR + ABX	FICI	Result
H37Rv	CUR	3.68 μg/mL	1.84 μg/mL	1	Additive
	INH	0.0625 μg/mL	0.03125 μg/mL		
	CUR	3.68 μg/mL	0.92095 μg/mL	0.75	Partial synergy
	RIF	0.125 μg/mL	0.0625 μg/mL		
MDR	CUR	7.36 μg/mL	1.84 μg/mL	0.75	Partial synergy
	AMK	0.5 μg/mL	0.25 μg/mL		
	CUR	7.36 μg/mL	1.84 μg/mL	0.75	Partial synergy
	MOX	0.0625 μg/mL	0.03125 μg/mL		

**Table 2:** Distribution of the animals used in the study.

Group	Number of Mice
H37Rv + NSS	24 (12 per time of euthanasia)
H37Rv + ABX	24 (12 per time of euthanasia)
H37Rv + ABX+ Curcumin	24 (12 per time of euthanasia)

**Table 3:** Gene expression was assessed using the following primer sequences.

Gene	Forward	Reverse
*GAPDH*	5′-CATTGTGGAAGGGCTATGA-3′	5′-GGAAGGCCATGCCAGTGAGC-3′
*TNF*	5′-GCCGAGAAAGGCTGCTTG-3′	5′-TGTGGCTTCGACCTCTACCTC-3′

## Data Availability

The data generated in this work are available upon direct request to the authors due to the department’s confidentiality policies.

## Abbreviations

The following abbreviations are used in this manuscript:

ABX	Antibiotics
AMK	Amikacin
ARRIVE	Animal Research: Reporting of In Vivo Experiments
BDNF	Brain-Derived Neurotrophic Factor
BSL-3	Biosafety Level 3
CFU	Colony Forming Unit
COX-2	Cyclooxygenase-2
DMSO	Dimethyl sulfoxide
FBS	Fetal Bovine Serum
FICI	Fractional Inhibitory Concentration Index
GAPDH	Glyceraldehyde-3-phosphate dehydrogenase
H&E	Hematoxylin and Eosin
IL-12	Interleukin-12
INCMNSZ	Instituto Nacional de Ciencias Médicas y Nutrición Salvador Zubirán
INH	Isoniazid
MDR	Multidrug-resistant
MIC	Minimum Inhibitory Concentration
MOI	Multiplicity of Infection
MOX	Moxifloxacin
*Mtb*	*Mycobacterium tuberculosis*
NFκB	Nuclear Factor kappa B
NSS	Normal Saline Solution
OADC	Oleic, Albumin, Dextrose, and Catalase
OD	Optical Density
PBS	Phosphate-Buffered Saline
PGE_2_	Prostaglandin E_2_
PPARγ	Peroxisome Proliferator-Activated Receptor Gamma
PYZ	Pyrazinamide
RAi	Red de Apoyo a la Investigación
RIF	Rifampicin
RPMI	Roswell Park Memorial Institute medium
RT-PCR	Reverse Transcription Polymerase Chain Reaction
SD	Standard Deviation
SDS	Sodium Dodecyl Sulfate
SEM	Standard Error of the Mean
TB	Tuberculosis
TNF	Tumor Necrosis Factor
XDR	Extensively drug-resistant
